# Women and Infectious Disease—Chronic Disease Interactions^1^

**DOI:** 10.3201/eid1011.040623_14

**Published:** 2004-11

**Authors:** Siobhán O'Connor, Sheila K. West, Breyette Lorntz, Frank Vinicor, Cynthia Jorgensen

**Affiliations:** * Centers for Disease Control and Prevention, Atlanta, Georgia, USA;; † Johns Hopkins University, Baltimore, Maryland, USA;; ‡ University of Virginia, Charlottesville, Virginia, USA

**Keywords:** developmental delays, children's health, chronic disease, infectious disease, Chlamydia trachomatis (trachoma), parasitic infections, women's health, diabetes, blindness

Relationships between infectious and chronic diseases are complex and diverse. Chronic infection, inflammation and immunity, malignant transformation, and other processes can cause progression to chronic disease. Sudden permanent deficits or secondary sequelae of events precipitated by infection can occur. Each point in time from acute infection to chronic outcome represents an opportunity to minimize infectious and chronic diseases. Women are disproportionately affected by recognized preventable chronic outcomes such as trachoma. Women are needed to help prevent childhood infections with long-term developmental sequelae. Chronic diseases can alter risk for infection, and infection may determine chronic disease complications or death.

## Chronic Ocular *Chlamydia trachomatis* (Trachoma)

Trachoma from ocular *C. trachomatis* infection accounts for one fourth of the >80% preventable or curable blindness in many countries. The blinding complications of trachoma disproportionately affect women, and its prevalence in disease-endemic regions is high. The cycle is vicious, from facile transmission with repeated infections, often involving severe inflammation, which promotes scarring and can lead to disabling trichiasis and blindness. Severe scarring and trichiasis increase with age, the result of repeated infections in childhood and young adulthood.

Trachoma is a community disease, clustered within families and neighborhoods. Caring for children, who are the reservoir of infection within these communities, increases women's risk for trachoma. The World Health Organization has endorsed a multipronged trachoma control strategy as part of the resolution for the Global Elimination of Blinding Trachoma by 2020. The strategy consists of surgery for trichiasis to decrease avoidable blindness, administration of antimicrobial drugs to reduce the community pool of infection, face washing to interrupt transmission, and environmental change to reduce transmission. Mass treatment alone, with single dose azithromycin or topical antimicrobial drugs for several weeks, can result in infection and trachoma reemergence from untreated and inadequately treated community members. The full intervention strategy is recommended, including hygiene activities and environmental improvement, to sustain reduction of trachoma in affected communities.

## Early Childhood Enteric and Parasitic Infections: the Role of Women

Worldwide, ≈2.5 million children die each year from enteric infections. We have shown long-term effects from nonfatal early childhood diarrhea that include growth shortfalls, fitness impairment, cognitive impairment, and poor school functioning in a longitudinal cohort of poor children with complete diarrhea surveillance from 0 to 2 years of age and quarterly evaluations from age 2. The detailed survey included enteric parasitic and bacterial infections. Five years later, early childhood diarrhea from 0 to 2 years of age correlated with impaired physical fitness and cognition; diarrhea negatively correlated with height-for-age Z-scores taken at 2 to 7 years old. The number of diarrhea episodes at 0 to 2 years negatively correlated with impaired cognitive function and decreased semantic and phonetic fluency, even when adjusted for maternal education, income, and birth size; significantly poorer school functioning was independent of maternal education, socioeconomic status, other illnesses, and stunting. Possible biologic explanations include a cycle of malnutrition and diarrhea and adverse effects on brain development. Early childhood diarrhea might double the disability-adjusted life-year for global diarrhea. Engagement of mothers and women is crucial to early childhood diarrhea prevention.

## Worldwide Impact of Infection on Type 2 Diabetes

The relationships between women, infections, and type 2 diabetes mellitus create a complex triangle. Insulin resistance followed by relative insulin deficiency typifies type 2 diabetes mellitus. Type 2 diabetes prevalence is increasing worldwide, affecting millions of women. Rates in the United States approach 8%–10% of adults, with lifetime risk 1/3, but this risk is 1/2 for Hispanic females and 2/5 for African-Americans. Obesity is the major contributor, but the aging population also contributes. Primary prevention focuses on improved screening and diagnosis and access to and utilization of quality health care. Risk for serious infections increases with poor diabetes control, but more complex relationships between infection and type 2 diabetes are now appreciated. For example, in U.S. NHANES III national surveillance, type 2 diabetes increases among people with hepatitis C virus infection. Insulin resistance and beta-cell failure occur; the HCV genotype may be important. Neuropathic and vascular foot ulcers with skin breakdown, predisposed to infection and osteomyelitis, remain a problem. The importance of asymptomatic bacteruria (infection vs. non-pathogen) in diabetic women is poorly understood, and alone, probably should not be treated with antibiotics. Viruses may also affect diabetics. Influenza is always a concern. Less clear, several severe acute respiratory syndrome reports noted diabetes among 1%–37% of critically ill (*1**–**3*). These examples exemplify dynamic diabetes-infection interactions. Obesity prevention, diabetes control, and good infection prevention measures may affect women and men at risk for and with type 2 diabetes.

## References

[1] Booth CM, Matukas LM, Tomlinson GA, Rachlis AR, Rose DB, Dwosh HA, Clinical features and short-term outcomes of 144 patients with SARS in the greater Toronto area. JAMA. 2003;289:2801–9. 10.1001/jama.289.21.JOC30885 12734147

[2] Fowler RA, Lapinsky SE, Hallett D, Detsky AS, Sibbald WJ, Slutsky AS, Critically ill patients with severe acute respiratory syndrome. JAMA. 2003;290:367–73. 10.1001/jama.290.3.367 12865378

[3] Tsang KW, Ho PL, Ooi GC, Yee WK, Wang T, Chan-Yeung M, A cluster of cases of severe acute respiratory syndrome in Hong Kong. N Engl J Med. 2003;348:1977–85. 10.1056/NEJMoa030666 12671062

